# Supplementary material for: Bifidobacterium castoris strains isolated from wild mice show evidence of frequent host switching and diverse carbohydrate metabolism potential
Source: ISME Commun. 2022 Feb 25;2:20. doi: 10.1038/s43705-022-00102-x (PMC9723756; doi:10.1038/s43705-022-00102-x)
Supplement: Supplementary file 1 — Supplementary Figure & Table Legends [file 43705_2022_102_MOESM1_ESM.docx]

**Supplementary Figure and Table legends**

**Supplementary Figure 1**. Proportional increase (%) in the growth of *B. castoris* isolates representative of the 12 strains identified in this study in mMRS supplemented with either 0.5% normal maize starch (NMS) (turquoise) or chitosan (grey) relative to unsupplemented mMRS represented as colony forming units per ml (CFU/ml). *B. longum* NCIMB 8809 was used as negative control.

**Supplementary Figure 2.** Functional classification of proteins predicted to be horizontally acquired by *B. castoris* isolates according to COG categories based on the available eggNOG annotation. The eggNOG annotation was available for 49.22±5.69% of putative horizontally acquired genes per genome, on average.

**Supplementary Table S1.** Animal host metadata, details on animal hosts positive for *Bifidobacterium*, genomic data on the isolates in this study, publicly available genomes used in this study (separate tabs).

**Supplementary Table S2.** Average nucleotide identity between *Bifidobacterium* isolates recovered in this study.

**Supplementary Table S3.** List of core and unique genes identified in *B. castoris* pangenome.

**Supplementary Table S4.** Markdown report from R code used for data analysis with 'ape', 'vegan' and 'indicspecies'.

**Supplementary Table S5.** Summary of COG categories identified in *B. castoris* isolates. COG categories legend, COG categories identified in B. castoris strains (separate tabs).

**Supplementary Table S6.** Summary of CAZymes identified in *B. castoris* strains. GH: glycosyl hydrolase, GT: glycosyl transferase, CE: carbohydrate esterase, CBM: carbohydrate-binding module.

**Supplementary Table S7.** Multilevel pattern analysis of association between glycosyl hydrolase repertoires of the 12 *B. castoris* strains and their respective hosts using the point biserial correlation coefficient.

**Supplementary Table S8.** Protein homology between *B. castoris* sequences and those previously identified as *Bifidobacterium* *eps*-key components, based on comparison with *eps3* and *eps4* clusters present in *B. animalis* subsp. *lactis* Bl12 and *B. pseudolongum* subsp. *globosum* LMG 11569T (BLASTP, e-value 1e-5, filtered based on 50% identity and 50% coverage).

**Supplementary Table S9.** Summary of genes predicted to be horizontally acquired by *B. castoris* strains, for which functional annotation was available.
