# Supplementary material for: Bifidobacterium castoris strains isolated from wild mice show evidence of frequent host switching and diverse carbohydrate metabolism potential
Source: ISME Commun. 2022 Feb 25;2:20. doi: 10.1038/s43705-022-00102-x (PMC9723756; doi:10.1038/s43705-022-00102-x)
Supplement: Supplementary file 2 — Supplementary Figure 1 [file 43705_2022_102_MOESM2_ESM.pdf]

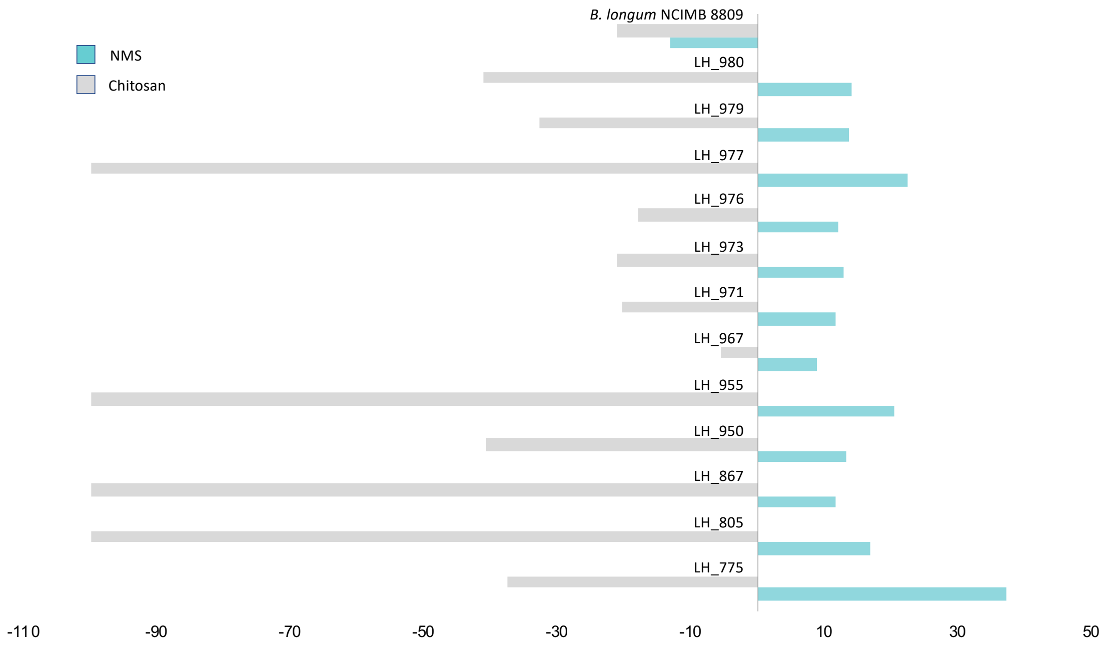

Supplementary Figure 1. Proportional increase (%) in the growth of *B. castoris* isolates representative of the 12 strains identified in this study in mMRS supplemented with either 0.5% normal maize starch (NMS) (turquoise) or chitosan (grey) relative to unsupplemented mMRS represented as colony forming units per ml (CFU/ml). *B. longum* NCIMB 8809 was used as negative control.
