# Supplementary material for: Bifidobacterium castoris strains isolated from wild mice show evidence of frequent host switching and diverse carbohydrate metabolism potential
Source: ISME Commun. 2022 Feb 25;2:20. doi: 10.1038/s43705-022-00102-x (PMC9723756; doi:10.1038/s43705-022-00102-x)
Supplement: Supplementary file 3 — Supplementary Figure 2 [file 43705_2022_102_MOESM3_ESM.pdf]

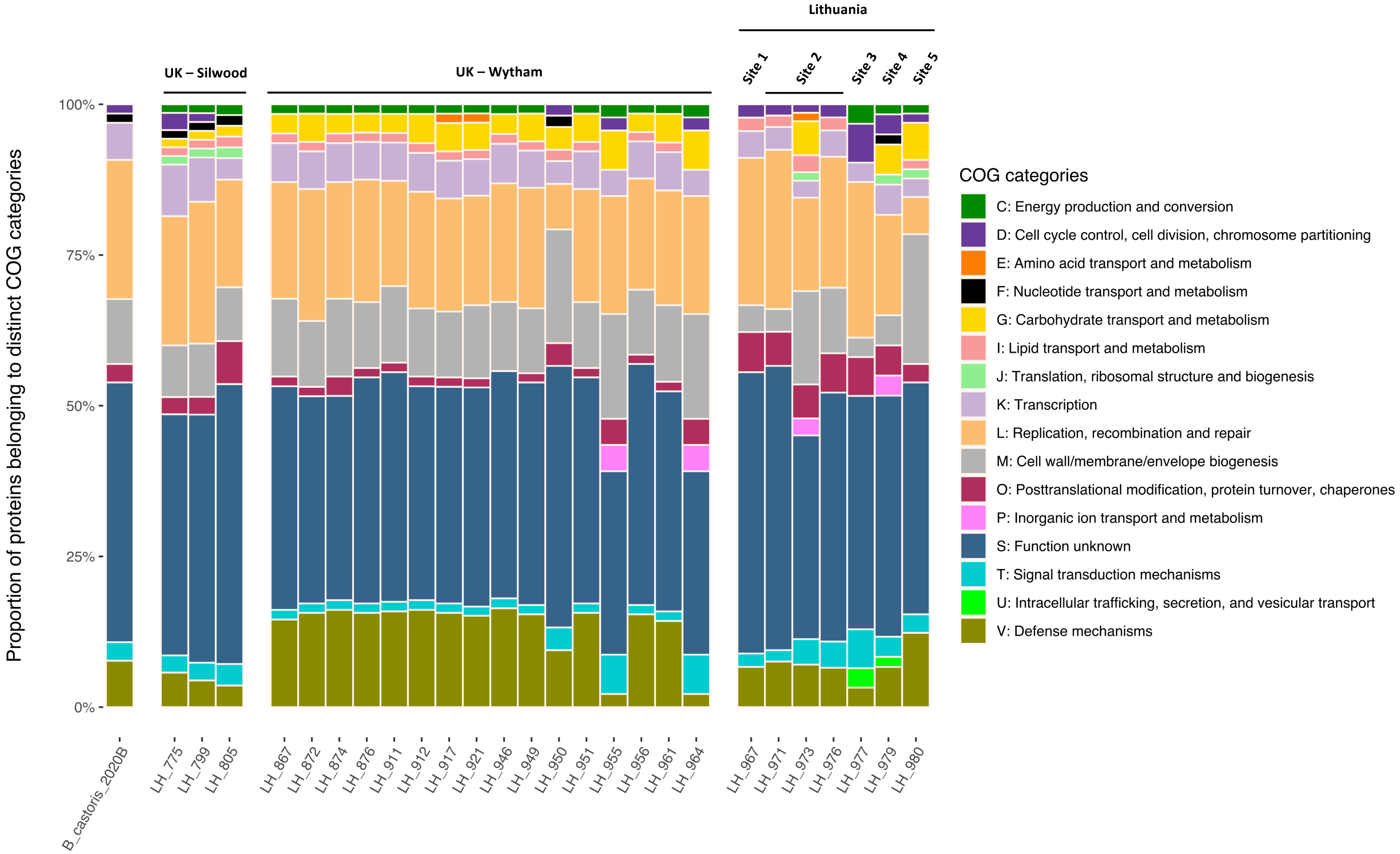

Supplementary Figure 2. Functional classification of proteins predicted to be horizontally acquired by *B. castoris* isolates according to COG categories based on the available eggNOG annotation. The eggNOG annotation was available for 49.22±5.69% of putative horizontally acquired genes per genome, on average.
